# Repeated exposure of fluazinam fungicides affects gene expression profiles yet carries no costs on a nontarget pest

**DOI:** 10.1111/1744-7917.13013

**Published:** 2022-03-25

**Authors:** Shahed Saifullah, Aigi Margus, Maaria Kankare, Leena Lindström

**Affiliations:** ^1^ Department of Biological and Environmental Science University of Jyväskylä Jyväskylä Finland

**Keywords:** behavior, fluazinam, gene expression, *Leptinotarsa decemlineata*, metabolic detoxification, nontarget animal

## Abstract

Fungicides are used to control pathogenic fungi of crop species, but they have also been shown to alter behavioral, life history and fitness related traits of nontarget insects. Here, we tested the fungicide effects on feeding behavior, survival and physiology of the nontarget pest insect, the Colorado potato beetle (CPB) (*Leptinotarsa decemlineata*). Feeding behavior was studied by a choice test of adult beetles, which were allowed to choose between a control and a fungicide (fluazinam) treated potato leaf. Larval survival was recorded after 24 and 72 h exposure to control and fungicide‐treated leaves with 2 different concentrations. The adults did not show fungicide avoidance behavior. Similarly, survival of the larvae was not affected by the exposure to fungicides. Finally, to understand the effects of fungicides at the physiological level (gene expression), we tested whether the larval exposure to fungicide alter the expression of 5 metabolic pathway and stress associated genes. Highest concentration and 72‐h exposure caused upregulation of 1 cytochrome P450 (*CYP9Z14v2*) and 1 insecticide resistance gene (*Ldace1*), whereas metabolic detoxification gene (*Ugt1*) was downregulated. At 24‐h exposure, highest concentration caused downregulation of another common detoxification gene (*Gs*), while both exposure times to lowest concentration caused upregulation of the *Hsp70* stress tolerance gene. Despite these overall effects, there was a considerable amount of variation among different families in the gene expression levels. Even though the behavioral effects of the fungicide treatments were minor, the expression level differences of the studied genes indicate changes on the metabolic detoxifications and stress‐related pathways.

## Graphical abstract

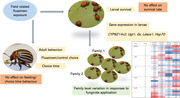

## Introduction

Fungal pathogens of crops are considered one of the most economically significant phyto‐pathogens that can be a severe threat to food security (Pennisi, 2010; National Academy of Sciences, 2011). Agricultural fungicides control fungal diseases of crops and as the severity of fungal diseases has increased over the past few decades (Fisher *et al*., 2012) so has fungicide usage. In Europe, fungicides are one of the most widely used plant protection chemicals (41.76% in 2015: Eurostat, 2020) and it is likely that fungicide residuals are also present in our environment (air or water) and even in food (Woodrow *et al*., 1995; Cabras *et al*., 1997; Caldas *et al*., 2001; Kreuger *et al*., 2010; EFSA, 2016) as fungicide exposed animals and plant materials can be found in many food chains (Walorczyk *et al*., 2013; Mu *et al*., 2016). Presence of fungicides in food may in turn have negative impacts on the health of consumers. However, the impact of fungicide on animals may vary depending on the active ingredient and its pattern of exposure (Piel *et al*., 2019). In general, fungicides are designed to target essential cellular or physiological processes in fungi (FRAC, 2021), but they may also target common cellular organelles in other organisms, which are similar in fungi and in eukaryotes (Maltby *et al*., 2009). Several studies have shown the potential negative impacts of fungicides on many organisms, such as fish, pollinators, arthropods and so forth (Wightwick *et al*., 2010; Elskus, 2014; Shi *et al*., 2018). Indeed, excessive use of agricultural fungicides can induce costs to species living in the agro‐environment.

Toxicity information of many fungicide classes is mostly available for fish, rats and mice, although these organisms are unlikely to be regularly exposed to fungicides (Oruc, 2010; Rouabhi, 2010). Nontarget organisms, such as plants, insects, micro‐organisms, aquatic animals and birds that live near to the fungicide application sites, are common nontarget species (Zaller & Brühl, 2019). Moreover, the effects of many commonly used fungicides are still unknown for many likely nontarget organisms such as pollinators or pest insects in the agricultural settings (see Campbell *et al*., 2016; Clements *et al*., 2018a). Fungicides can be applied once or multiple times in the field depending on the severity of the fungal diseases (Reilly *et al*., 2012) and hence species living in the crop fields can be exposed to fungicides for both short and extended periods of time. Effects of the fungicide can also vary depending on the duration of the exposure of the nontarget species to the fungicide (Damalas & Eleftherohorious, 2011). Eco‐toxicological data for fungicides are mostly from short‐term studies focused on the acute toxic effect on mortality, but chronic exposure data and sublethal effects of fungicides on other fitness related traits are also important to understand (Elskus, 2014; Sancho *et al*., 2016). Fungicide concentrations and the mode of exposure found in agricultural fields may have both acute toxic effects after 1, or chronic effects (on fitness related traits) after multiple exposures (Wightwick *et al*., 2010; Reilly *et al*., 2012).

Several studies have reported the effects of fungicides on multiple fitness and life history related traits in nontarget organisms, including fish, amphipods, moths, beneficial and pest insects and so forth (Biggs & Hagley, 1988; Michaud, 2001; Adamski *et al*., 2011; Vu *et al*., 2016, 2017). For instance, in *Agrotis segetum* (turnip moth), fungicide (mancozeb) negatively affected the egg count and development of the larvae, but the larval survival remained unaffected (Adamski *et al*., 2011). Another fungicide (boscalid) reduced both the survival and reproduction (the number of offspring) in a marine arthropod (*Allorchestes compressa*) (Vu *et al*., 2016). Moreover, 2 other fungicides, (propiconazol and chlorothalonil) decreased larval survival and egg hatching success in the Japanese beetle (*Popillia japonica*) (Obear *et al*., 2016) and these 2 chemicals also decreased the activity of detoxification related enzymes in the larvae. In the Colorado potato beetle (CPB) (*Leptinotarsa decemlineata*), chlorothalonil and boscalid altered the activity of glutathione S‐transferase enzyme and multiple genes involved in metabolic detoxification pathways (Clements *et al*., 2018a). A study with zebra fish (*Danio rerio*) showed that a commonly used fungicide (fluazinam), can also alter the expression of stress‐related genes (Wang *et al*., 2018). Therefore, the effects of fungicides on any nontarget species should be studied at multiple levels ranging from behavior to gene expression levels.

Fungicides target fungal cell membrane components or essential cellular functions, like nucleic acid or protein synthesis, cell division, signal transduction or respiration (Yang *et al*., 2011; FRAC, 2021). Fungicides that have target sites in the mitochondrial respiratory system of the fungi are one of the most popular classes due to their broad‐spectrum anti‐fungal activity and low toxicity to nontarget mammals (EFSA, 2016; FRAC, 2021). Studies with respiratory inhibitor fungicides have shown they do not cause acute toxicity to mammals, birds and bees but are highly toxic to fish, marine and freshwater invertebrates (Tomlin, 2000; Elskus, 2014). Fluazinam, an oxidative phosphorylation (OXPHOS, an essential stage in the mitochondrial adenosine triphosphate [ATP] synthesis reaction) inhibitor fungicide, is commonly used in various crop fields to control a broad range of fungal diseases. These include molds (e.g., *Botrytis cinerea*), stem and root rot (*Sclerotinia sclerotiorum*), and potato blights (e.g., *Phytophthora infestans* and *Alternaria solani)* (Kalamarakis *et al*., 2000; Runno‐Paurson *et al*., 2015; Schepers *et al*., 2018). In the fungal cell, the mode of action of fluazinam involves the inhibition of ATP synthase enzyme, that is, it blocks ATP production (Vitoratos, 2014). If fluazinam can also affect mitochondria and/or the respiration process in other organisms, this could explain the negative effects of such OXPHOS inhibitor fungicides in nontarget organisms, as complete or partial inhibition of ATP can compromise growth, development, activity and immune function (Campbell *et al*., 2016; Wang *et al*., 2018). Earlier studies have indicated that unlike in fungi (where it targets ATP synthase), fluazinam does not have specific target sites in nontarget species, but it impairs to some degree energy production (Guo *et al*., 1991; Lee *et al*., 2012; Wang *et al*., 2018). In addition, fluazinam toxicity may vary between different species due to the difference in metabolism and excretion process of the fungicide in different organisms (Guo *et al*., 1991). Some animals may also be more susceptible to fungicide than others, due to their physiology or behavior (Oruc, 2010). However, very little information exists on the effects of fungicides on invertebrates that frequently come into contact with these chemicals in agricultural fields.

During the growing season, potato fields can be heavily infested with early and late blight (*A. solani* and *P. infestans*) which are controlled by fungicides, including fluazinam (FRAC MoA C5) (Reilly *et al*., 2012; Schepers *et al*., 2018) and nontarget species like arthropods living in the agricultural field can also be affected. As the most likely nontarget species of crop fields, pest insects are particularly interesting, because they can be repeatedly affected by fungicides. Even though the effects of few classes of fungicides, such as boscalid, chlorothalonil and mancozeb have been studied in some pest insects, information about the effects of many common fungicides on nontarget pest species are still unavailable (Adamski *et al*., 2011; Patterson & Alyokhin, 2014; Obear *et al*., 2016; Clements *et al*., 2018a). Moreover, the effects of the most commonly used class of respiratory inhibitor fungicide, that is, OXPHOS inhibitors, of a specific crop, like potato, are still unknown for its most likely nontarget pest species, CPBs.

The CPB is the most harmful insect pest of potato (*Solanum tuberosum*) (Alyokhin *et al*., 2008). As it is an invasive pest species, chemical insecticides are used to control its occurrence worldwide, and hence many CPB populations have developed resistance to various insecticides (Huseth *et al*., 2014; Mota‐Sanchez & Wise, 2021). Besides the insecticides that are applied frequently in the fields, other chemical inputs may also play a role in the development of insecticide resistance in the species (Clements *et al*., 2018a). One of the important factors can be the cross‐resistance between insecticides and fungicides that may facilitate the rapid development of resistance in a population. Earlier studies have shown that fungicides can negatively affect larval survival and development, and can also activate metabolic detoxification related pathways similar to those affected by the insecticide treatments in CPB (Patterson & Alyokhin, 2014; Clements *et al*., 2018a, 2018b). However, the effects of the OXPHOS inhibitor fungicides, like fluazinam, on CPB have not been investigated. Beetles are most likely exposed to fluazinam in the potato field multiple times during either their larval and/or adult stages since the fungicide is applied throughout the growing season (Schepers *et al*., 2018). If fluazinam affects the respiratory system of the beetle, it may affect the survival or physiology of the larvae (by altering enzyme and gene activity). Exposure to fungicide, as often shown in insecticide exposure, may also induce behavioral responses (avoidance of the fungicide‐treated leaves) in the adult beetles (e.g., feeding behavior). Therefore, our aim of this study was 2 fold: to understand the effects of fluazinam, on (i) the adult and (ii) larval stage of the CPB. We tested the feeding behavior of the CPB adults under 3 field related concentrations of the fluazinam used in Finland and in other European countries (EU Pesticides database, 2021). First, we tested whether the fungicide is aversive to the beetles and whether this aversion is related to the level of fungicide concentrations on the treated potato leaves by measuring feeding behavior. Further, we checked whether there are differences in the feeding behavior between the sexes. Second, we investigated the direct effects of fungicide on larval performance by measuring larval survival under different fungicide treatments (i.e., control, 24 and 72‐h exposure to 0.25 and 0.66 mg/L of fluazinam). Finally, we investigated gene expression level differences between different treatments using 3 metabolic detoxification related genes (*CYP9Z14v2*, *Ugt1*, and *Gs*), 1 insecticide resistance gene (*Ldace1*), and 1 stress tolerance gene (*Hsp70*).

## Materials and methods

### Study species

We used the laboratory population of the CPB originally collected from Vermont, USA (44°43′ N, 73°20′ W) in 2010. To maintain the lab population, we mated the field collected adult beetles in laboratory conditions and new adults of the next generation were overwintered in controlled chambers at 5 °C (detailed rearing conditions are described in Lehmann *et al*., 2014; Margus, 2018; Margus *et al*., 2019). We used the 7th generation of the adults for the behavior trials (choice and food consumption experiments, summer 2017) and larvae from the 8th generation for the survival and gene expression experiments (summer 2018).

### Fungicide choice experiment on adults

We chose randomly 133 adults (62 females and 71 males) from 24 families of the CPB for the fungicide choice experiment, where we allowed each individual to choose from either control or fungicide‐treated leaves (Table 1). Commercial fungicide product, Shirlan (Syngenta Crop Protection AG, Switzerland) which contains fluazinam (500 g/L) as active ingredient was used for the experiment. We used half of the lowest (0.12 mg/L), lowest (0.25 mg/L) and highest (0.66 mg/L) field recommended concentration of fluazinam for the experiment. Before the experiment the adult beetles were weighed (± 1 mg) (AM100, Mettler, Columbus, OH, USA) and then randomly divided into 3 fungicide concentration groups: 0.12 mg/L (*N* = 42), 0.25 mg/L (*N* = 46) and 0.66 mg/L (*N* = 45). We allowed beetles to choose from 2 potato leaf discs (1.7 cm in diameter), which were either dipped in distilled water (control) or in the different fungicide solutions. The 2 leaf discs were offered to the beetles on a Petri dish (9 cm in diameter). The adult beetle was put on its back in the center of the Petri dish and the beetle's choice was counted when it started eating the leaf discs. The choice the beetles made (control or fungicide) and the time it took to choose the leaf were recorded. Each of the adults was allowed to choose 3 times between a new set of treated and control leaf discs.

**Table 1:** Experimental setup for feeding behavior, sample size and descriptive statistics by different concentrations and sexes

	Fungicide concentration		
	0.12 mg/L	0.25 mg/L	0.66 mg/L
Females, *n*	19	22	21
First choice control: fungicide	10 : 9	12 : 10	9: 12
Average choice (*SEM*)	0.56 (± 0.1)	0.47 (± 0.1)	0.49 (± 0.1)
Time in first trial (*SEM*), min	8.16 (± 1.8)	18.73 (± 3.9)	15.57 (± 3.3)
Average time (*SEM*), min	10.78 (± 1.7)	12.53 (± 1.6)	14.17 (± 1.6)
Males, *n*	23	24	24
First choice control: fungicide	14 : 9	12 : 12	12 : 12
Average choice (*SEM*)	0.46 (± 0.1)	0.51 (± 0.1)	0.51 (± 0.1)
Time in first trial, (*SEM*) min, by type	control 15.03 (± 2.5): fungicide 22.36 (± 3.2)		
Average time (*SEM*), min	14.09 (± 2.1)	11.17 (± 1.9)	13.19 (± 2.2)

First choice refers to which treated leaf the beetles chose first, average choice (± *SEM*) to how beetles chose on average (random choice = 0.5), the time it took the first leaf for females, by different concentrations, and for different leaf types for males and finally the average time (± *SEM*) in min it took for beetles to choose the leaves.

### Larval survival experiment

To test the direct effect of 2 field related fluazinam concentrations (0.25 mg/L and 0.66 mg/L) and treatment types (24 and 72‐h exposure) on larval survival, we exposed the 2nd instar (i.e., 2–3‐d‐old) larvae (*N* = 875) from 12 different families either to control (distilled water) or to a fluazinam‐treated leaf disc. We had 5 treatments in total (control, 24‐h exposure of 0.25 and 0.66 mg/L and 72‐h exposure of 0.25 and 0.66 mg/L of fluazinam). We took 15 larvae from each family and randomly divided them into 5 treatment groups where each group had 3 larvae. We repeated each of the families 4–5 times for each treatment group. For the exposure, we dipped a leaf into a fungicide solution and placed them into a randomly chosen well (36 mm in diameter) of a 6‐well falcon plate (127 mm in length). In the 24‐h exposure treatments, we exposed the larvae once to the treated leaf at the beginning of the experiment and subsequently gave beetles fresh leaves after 24 and 48 h. For the 72‐h exposure, we exposed the larvae to the new fungicide‐treated leaf 3 times (at the beginning, after 24 h and after 48 h). We recorded the 72‐h survival and snap froze living larvae with liquid nitrogen and stored them at −80 °C for RNA extractions.

### Target gene selection

We chose to test the effects of fungicide treatment on the expression levels of 5 candidate genes, which have been previously associated with metabolic detoxification (3 genes), insecticide resistance (1 gene), and stress tolerance (1 gene) in CPB (detailed below).

CPB has shown to have metabolic resistance against carbamate, organophosphate and pyrethroid insecticides (reviewed in Kaplanoglu, 2016). A common enzyme group involved in the metabolic detoxification is cytochrome P450 (CYPs; Li *et al*., 2007; Feyereisen, 2012). *CYP9* genes of this family has been found to be associated with resistance to pyrethroids and organophosphate insecticides in several different insects, like in *Bombyx mori* (silkworm) and *Rhynchophorus ferrugineus* (weevil beetle) (Zhao *et al*., 2011; Antony *et al*., 2019) as well as in CPB (Clements *et al*., 2016; Zhu *et al*., 2016). For this study, we selected detoxification gene *CYP9Z14v2*, which has been shown to be upregulated in the neonicotinoid resistant individuals of CPB (Zhang *et al*., 2008; Kaplanoglu *et al*., 2017). We also chose another metabolic detoxification related gene, *Uridine diphosphate glycoronosyltransferase 1* (*Ugt1*) which is associated with the metabolic detoxification of insecticides in CPB (Kaplanoglu *et al*., 2017; Clements *et al*., 2018b). In addition, we used *Glutathione synthetase* (*Gs*) gene, which is known to be related to neonicotinoid resistance in CPB (Clements *et al*., 2016, 2017) and *acetylcholine esterase1* gene (*Ldace1*), with high expression levels associated with resistance against organophosphate and carbamate insecticides in CPB (Revuelta *et al*., 2011; Margus *et al*. 2021). Finally, we measured the expression levels of a heat shock protein gene, *Hsp70*, which is an early marker of stress associated with the respiration process and temperature shock (Lee *et al*., 2012; Chen *et al*., 2014; Wang *et al*., 2018).

Primers for 4 of the above‐mentioned target genes, *CYP9Z14v2*, *Gs*, *Ldace1* and *Hsp70* and for 2 reference genes used in quantitative real‐time polymerase chain reaction (qPCR), ribosomal protein S18 gene (*RpS18*) and 50S ribosomal protein *L13e* gene (*L13e*) were collected from published studies (Table 2). In addition, we designed primers for *Ugt1* gene using the annotated transcriptome of the CPB (DDBJ/EMBL/GenBank accession: GEEF00000000, Clements *et al*., 2016) with programs Net Primer (http://www.premierbiosoft.com/netprimer/) and Primer3 (version 0.4.0, http://bioinfo.ut.ee/primer3‐0.4.0/).

**Table 2:** Primer sequences used in the quantitative real‐time polymerase chain reaction analysis

Target genes	E%	*R* ^2^	Forward and reverse primers (5′–3′)	Product size (bp)	Response (treatment/pesticide)
*CYP9Z14v2*	100.4	0.998	F: ACCAATGCGTTTCAATCCCG R: CCAACCCGAATGGCAAATAAG	82	Environmental stress and host plant response^1^
Ugt1*	90.3	0.996	F: CGCTGAAGAGTTTGGGCTGT R: TCAGATCGGGACAGTGAGGAA	270	Chlorothalonil and imidacloprid^2^
*Gs*	97.8	0.999	F: CAGAGCAGGGTATGAACCTAATC R: CCAGCCAAGTGATACTGAATCG	114	Chlorothalonil^3^
*Ldace1*	96.7	0.998	F: CGCCGAGTTACAAAATACCC R: TAGCGTTTCCATCCAATTCC	124	Organophosphate^4^
*Hsp70*	101.8	0.999	F: GACGAGAAGCAAAGGCAAAG R: TGAGCGGTCTGTTTGATCTG	77	Heat shock^5^
*L13e*	91.3	0.999	F: TATTCACCAGCCATCCATCA R: GCGTCCTTCACTCTCTTTGC	138	Reference gene^6^
*RpS18*	91.8	0.999	F: TCCTCGCCAGTACAAAATCC R: ACACGGAGACCCCAGTAGTG	174	Reference gene^7^

Primers were either obtained from published studies or designed for the current study. Primer amplification efficiency (E%) and *R*‐squared (*R* ^2^) values are given for each of the primer pairs. Response gives the information about the stress conditions under which the gene was tested in the published study.

^1^ Zhang *et al*. (2008), ^2^Clements *et al*. 2018a, ^3^Clements *et al*. (2018b), ^4^Chen *et al*. (2014), ^5^Revuelta *et al*. (2011), ^6^Youcum *et al*. (2009), ^7^Pauchet *et al*. (2009). *Primers were designed from the published transcriptome data.

### Extraction of RNA, cDNA synthesis and gene expression analyses with qPCR

For gene expression analysis we randomly chose 6 unrelated CPB families out of 12 that were exposed to 5 fungicide treatments (control, 24 and 72‐h exposure to 0.25 and 0.66 mg/L of fluazinam). We then randomly took 5 larvae (out of 15 treated) from each of the treatments for each the 6 families for RNA extractions. We had in total 150 larvae from 5 treatment groups (5 larvae for 6 families/treatment).

Total RNA was extracted from single larvae with TRI Reagent (Sigma‐Aldrich, St. Louis, MO, USA) and RNeasy RNA isolation kit (Qiagen, Hilden, Germany). Concentration and the integrity of the RNA were measured with TapeStation (Agilent 2200, Santa Clara, CA, USA). The concentration of the RNA from each of the single larval samples were then normalized to 400 and 20 ng/μL and were used for complementary DNA (cDNA) synthesis using iScript cDNA Synthesis Kit (Bio‐Rad, Laboratories Inc., Hercules, CA, USA) according to the manufacturer's protocol. qPCR reaction was performed with a total volume of 20 *μ*L by using 1 *μ*L of cDNA, 10 *μ*L of 2 × SYBR Green Supermix (Bio‐Rad Laboratories Inc.), 1 *μ*L of the forward (10 *μ*mol/L) and 1 *μ*L of the reverse (10 *μ*mol/L) gene‐specific primers, and 7 *μ*L of nuclease‐free water. qPCR reactions were run on a CFX96 instrument (Bio‐Rad) with the following temperature cycles: initiation at 95 °C for 3 min and 39 cycles of 10 s at 95 °C, 10 s at 56 °C and 30 s at 72 °C. Melting curves of the reactions to check their amplification purity were then measured at 65 °C to 95 °C. For each treatment group and family, we used 5 larvae (biological replicates) with 3 technical replicates and the final threshold value (Cq) was defined as a mean of the technical replicates that produced good quality data. Normalization of the qPCR data was done with ∆∆(Ct) normalization method (Pfaffl, 2001) with *RpS18* and *L13e* as reference genes (these genes had equal expression levels in all the compared samples, data not shown) using Bio‐Rad CFX Maestro 1.1 program. Efficiency of the genes was quantified using 2‐times dilution series with the same software. Statistical significance of the expression level differences between different treatments and family groups was analyzed with REST (http://rest.gene‐quantification.info/) software with 10 000 iterations and using real efficiency values.

### Statistical analyses

Choice (i.e., control or fungicide) made by the adult beetles was analyzed with Chi‐square test. To analyze the time it took for the beetles to choose treated and untreated leaves, we used the time (in minutes) taken to make the first choice as a dependent variable in analysis of covariance (ANCOVA) where fungicide treatment, first choice (control/fungicide) and sex were used as fixed factors, and the weight of the beetle as a covariate. We analyzed 72‐h larval survival with the binary logistic regression where the fungicide concentration treatment was used as a categorical factor and family as a random factor. Analyses were carried out using SPSS v.24 (IBM Corp. Armonk, NY, USA).

## Results

### Effects of the fungicide on feeding behavior

**Fig. 1:**
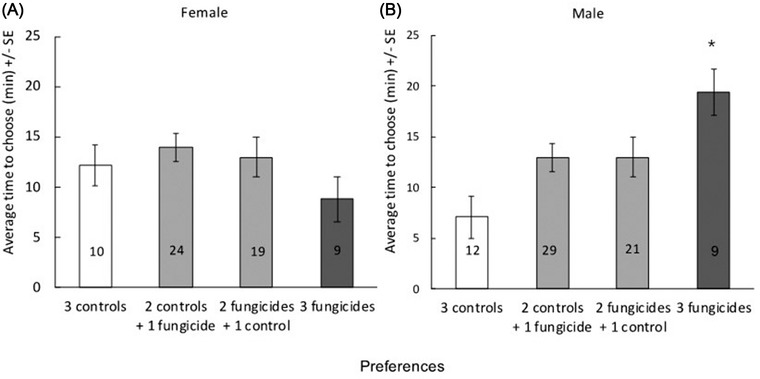
Average time (in min) taken to choose the 3 potato discs for (A) female and (B) male beetles with different preferences when all concentration treatments are combined. White bars indicate the beetles that chose only the control treated leaves, and light gray bars for the choice of both fungicide and control treated leaves after 3 trials. Dark gray bars indicate the beetles that chose only the fungicide‐treated leaf after 3 trials. Numbers in the bars show the number of individuals.

We offered both control and fungicide‐treated leaf discs to CPB adults and measured their feeding and response time behavior. We did not identify any general fungicide avoidance behavior since the beetles chose the fungicide‐treated leaves as likely as control leaves on their first choice (*χ* ^2^ = 0.957, *n* = 133, df = 2, *P* = 0.620). Moreover, there was no avoidance of fungicide‐treated leaves over the 3 trials (average choice, ANCOVA *F*_2,129_ = 0.053, *P* = 0.948). However, there was a difference in time it took for the female and male beetles to choose the treated leaves in the different fungicide treatments in the first trial (ANCOVA, *F*_2,130_ = 3.284, *P* = 0.041) and therefore the responses of the sexes were separately analyzed. There was a tendency that the fungicide treatment affected the time it took for females to take the first leaf disc (ANCOVA, *F*_2,55_ = 2.924, *P* = 0.066) and females seemed to be more hesitant to choose the leaf discs in the average (0.25 mg/L concentrations). However, for the males it appeared to take longer to take the fungicide‐treated leaf discs although the difference was not statistically significant (ANCOVA, *F*_1,64_ = 3.507, *P* = 0.067). When the average times for all trials were analyzed, these differences disappeared (females: fungicide treatment ANCOVA *F*_2,58_ = 1.329, *P* = 0.273; males: fungicide treatment ANCOVA *F*_2,67_ = 0.504, *P* = 0.606). When data were analyzed by individual choices there were altogether 93 adults that did not show any clear preference to fungicide or to control treated discs (Fig. 1). However, there were 40 beetles, that systematically chose only either the control (*n* = 22) or the fungicide discs (*n* = 18). Males (*n* = 9) that systematically chose fungicide discs were on average 12.9 min slower compared to males (*n* = 12) that chose the control discs (ANCOVA, *F*_1,20_ = 8.127, *P* = 0.011, Fig. 1B) whereas no such difference was observed in females (ANCOVA, *F*_1,18_ = 1.143, *P* = 0.301, Fig. 1A).

### Effects of fungicide treatments on the survival of CPB larvae

The overall 72‐h larval survival (*n* = 875) was very high (>95% for all groups, only 16 individuals died). The survival under 72‐h exposure to the highest fungicide concentration (0.66 mg/L, survival 95.5%) did not differ significantly from the other groups (control survival 100%; 24‐h exposure to 0.25 mg/L, survival 97.7%; 72‐h exposure to 0.25 mg/L, survival 98.3%; or from 24‐h exposure to 0.66 mg/L, survival 99.4 %) (Wald = 5.444, df = 4, *P* = 0.245). Finally, survival of the larvae did not differ among different families (Wald = 3.809, df = 11, *P* = 0.975).

### Expression of metabolic detoxification, insecticide resistance and stress tolerance genes under different fungicide concentrations in different beetle families

**Fig. 2:**
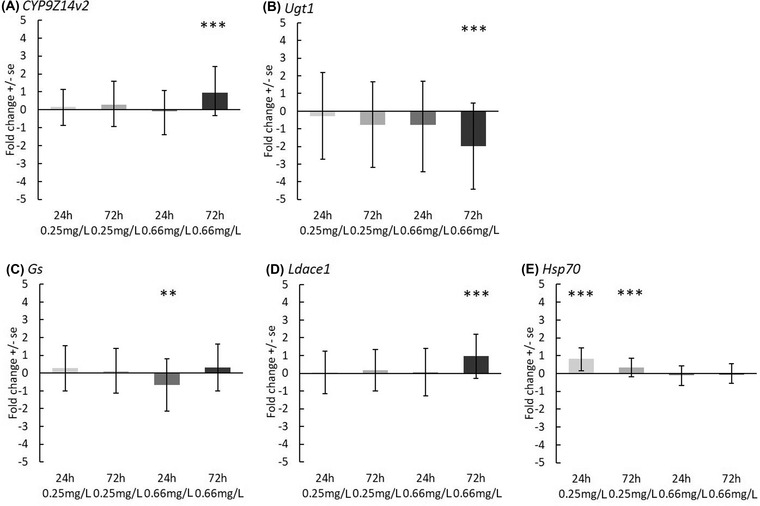
Fold change (log_2_ transformed ± *SE*) of the target genes, (A) *CYP9Z14v2*, (B) *Ugt1*, (C) *Gs*, (D) *Ldace1*, (E) *Hsp70*, in Colorado potato beetle (CPB) larvae under fungicide treatments (24 h of single and 72 h of repeated [3 times] exposure to 0.25 mg/L and 0.66 mg/L of fluazinam) when compared to control (nontreated) sample overall families. Expression levels of the study genes were normalized using *L13e* and *RpS18* reference genes. Significant differences between the control and the treatment groups are marked with asterisks (*P ≤ 0.05, **P ≤ 0.01, ***P = 0.001).

Long‐term exposure (when the larvae were exposed to fungicide for 72 h) to the highest fungicide concentration (0.66 mg/L) altered the expression of the investigated metabolic detoxification genes *CYP9Z14v2*, *Ugt1* and insecticide resistance gene, *Ldace1* (Fig. 2) in CPB larvae. The expression of *CYP9Z14v2* and *Ldace1* genes overall samples were significantly upregulated while that of *Ugt1* was downregulated. Moreover, short‐term (24 h) exposure to the highest fungicide concentration caused downregulation of *Gs* gene. Finally, both 24 and 72‐h exposure to the lowest field concentration of fungicide (0.25 mg/L) increased the expression of a stress tolerance gene *Hsp70* (Fig. 2).

**Fig. 3:**
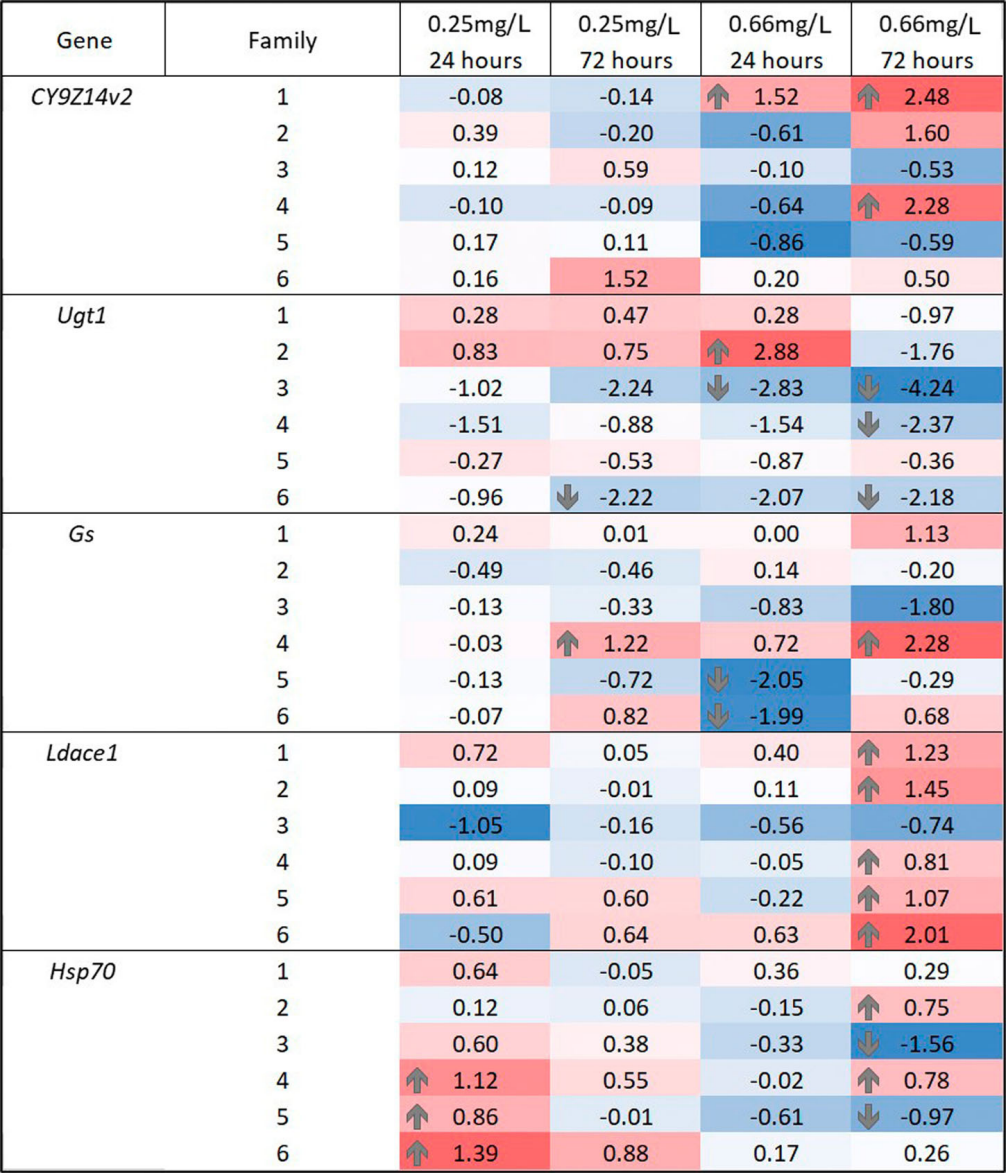
Fold changes (log_2_ transformed) of the 5 target genes (*CYP9Z14v2*, *Ugt1*, *Gs*, *Ldace1*, *Hsp70*) when compared to the control (nontreated) sample (*n* = 5) under 4 treatments (24‐h and 72‐h exposure to 0.25 mg/L and to 0.66 mg/L of fluazinam) in 6 Colorado potato beetle (CBP) families. Expression levels of the study genes were normalized using *L13e* and *RpS18* reference genes. Red color and positive values denote upregulation and blue and negative values indicate downregulation. Numbers from 1 to 6 indicate the 6 different families used in the study. Intensity of the color indicates the level of expression, and families with significant differences (collected from REST, see text for details) between the control and the treatment groups are marked with upward or downward arrows.

Due to the large variation in the gene expression levels over all samples, we separately analyzed expression changes in each of the CPB families (Fig. 3). There were no significant differences in the expression levels of *CYP9Z14v2* under 24 or 72‐h exposure to the lowest fungicide concentration in any of the families. However, the expression increased significantly in 1 family under 24‐h exposure, and in 2 families under 72‐h exposure to the highest fungicide concentration. The expression of *Ugt1* was unaffected in all the families under 24‐h exposure to the lower concentration while it showed significant downregulation in all except 2 families in the other treatments. Interestingly, in 1 family, *Ugt1* was significantly upregulated under 24‐h exposure to the higher fungicide concentration. Unfortunately, gene expression results of the third metabolic detoxification gene, *Gs*, are more difficult to interpret as this gene was significantly upregulated in one of the families under 72‐h exposure to both lower and higher concentrations while 2 other families showed significant downregulation in the 24‐h but not in the 72‐h treatment to the higher concentration. Expression levels of the insecticide resistance gene, *Ldace1*, were unaffected by both the 24 and 72‐h exposure to the lower concentration, as well as by the 24‐h exposure to the higher concentration. However, this gene was significantly upregulated in all except 1 family under the longer treatment of higher fungicide concentration when compared to control samples. Finally, expression of the stress tolerance gene, *Hsp70*, increased significantly in 3 families under the 24‐h exposure of the lower concentration. However, surprisingly, under the 72‐h exposure to the higher concentration, expression level of this gene both increased and decreased in different families while it remained unchanged under 72‐h exposure to the lower concentration and under 24‐h exposure to the higher fungicide concentrations in all families.

## Discussion

In agriculture, fungicides are important pesticides to reduce crop losses due to plant pathogens, but at the same time, their effects even at field related concentrations can extend to nontarget pest species. In this paper, we ran a series of experiments to test the effects of field related concentrations of fluazinam (a common fungicide used against the potato late blight) on the nontarget pest species, CPB. To get a comprehensive picture of the effects, we tested the fluazinam effects on beetles on 3 different levels: (i) at the behavioral (adult feeding behavior); (ii) at the individual (larval) fitness; and (iii) at the gene expression level.

In the adult behavioral tests beetles did not clearly avoid fungicide‐treated leaf discs, as they chose to eat both control and fungicide‐treated leaves at the same rate, and they did not even differentiate between different fungicide concentrations, suggesting that field related concentrations of fluazinam are not aversive to the beetles. Alternatively, it is possible that these concentrations were not high enough to be detected by the beetles. In aquatic ecosystems aversion behavior has been observed in juvenile zebra fish (*D. rerio*) and frog tadpoles (*Leptodactylus latrans*), which both avoided fungicide (pyrimethanil; FRAC MoA D1) contaminated water (Araújo *et al*., 2014a; 2014b). Although the results among different species and environments are not directly comparable (Elskus, 2014; Müller, 2018), the lack of choosing either of the leaves suggests that the CPB adults would be exposed to the fungicides in the potato field. However, there was individual level variation in the choosiness of the individuals (see Fig. 1), which may contribute to the exposure of fungicides in the field.

In addition to the adult behavior, neither of the field related fluazinam concentrations increased short‐term larval mortality in our study. Similar results have been observed before in zebrafish (Wang *et al*., 2018), although higher fluazinam concentrations disrupted mitochondrial bioenergetics and induced oxidative stress. Moreover, Clements *et al*., 2018a showed that boscalid fungicide (FRAC MoA C2) did not increase mortality directly, but that it delayed the CPB larval growth rate and larvae gained less mass compared to the control group, which also lead to a smaller size. Therefore, it is possible that delayed fitness effects related to fungicide exposure could also require longer experiments or fungicides used in combination with other stressors (see e.g., Cullen *et al*., 2019).

While there were no effects on adult behavior or larval mortality, the field related concentrations of fungicides still affected gene expression levels of the beetle larvae (see Shi *et al*., 2018; Fig. 2). Overall, out of the 5 genes tested, the smallest concentration affected only *Hsp70* gene expression, whereas the higher fluazinam concentration affected all the other 4 genes (Fig. 2), although there were big differences between different CBP families (Fig. 3). The fact that the higher concentration and repeated exposure increased gene expression the most, is a very general dose‐dependent finding (Wang *et al*., 2018) and suggests that even these low field related fluazinam concentrations may have metabolic costs for individuals. These costs may in turn have long‐term fitness consequences which were not observed here.

Out of the tested metabolic genes, *CYP9Z14v2* gene was upregulated and *Gs* and *Ugt1* were downregulated after exposure to the higher concentration of fungicide. Upregulation of cytochrome P450 (CYP) gene family could indicate that larvae were metabolizing fungicides like other toxic compounds (Terriere, 1984; Werck‐Reichhart & Feyereisen, 2000 see also Shang *et al*., 2020) such as insecticides (Clements *et al*., 2018a, 2018b) to minimize oxidative damages. Previously, upregulation of several *CYP* genes has been reported in CPB adults fed with fungicide (chlorothalonil, FRAC MoA M05) treated leaves (Clements *et al*., 2018a). Contrary to our findings, Clements *et al*. 2018a, 2018b) showed upregulation of *Ugt1* and *Gs* in CPB adults under fungicide treatments, suggesting that these genes are involved in the insect xenobiotic metabolism pathway. It is possible that the difference among the results is due to the exposure time or differences between the fungicides. However, both experiments suggest that these genes are important xenobiotic metabolic genes to study further.

The insecticide resistance associated gene, *Ldace1* (Fournier *et al*., 1992; Zhu & Gao, 1999; Clark *et al*., 2001), was upregulated under the highest concentration of fluazinam, suggesting that this chemical can act similarly like insecticides as high expression levels of *Ldace1* have been previously associated with the resistance against organophosphate and carbamate insecticides in CPB (Revuelta *et al*., 2011; Margus *et al*., 2021). On the other hand, if fluazinam slows down the growth of the larvae (see Clements *et al*., 2018a) it is possible that this difference between treatments is generated by the developmental differences among larvae as *Ldace1* expression goes down when the larvae grow (Revuelta *et al*., 2011). In contrast to our results, glyphosate‐based herbicides inhibited *Ldace1* expression in CPB larvae (Modesto & Martinez, 2010; Margus *et al*., 2019). The fact that both fungicides and herbicides can affect resistance associated genes underlines that even though fungicide effects are minor, as they can affect the same pathways as insecticides, they may also contribute to overall insecticide resistance.

Out of the studied genes, only *Hsp70* was upregulated on the lower fungicide concentration. HSPs perform as molecular chaperones that typically stabilize the structure and functions of the proteins in the cells under thermal stress conditions (Sun *et al*., 2014) or insecticide toxicity (Jing *et al*., 2013). Previously it has been shown that insecticide (imidacloprid) can increase the expression levels of the *Hsp70* gene of CPB under optimal temperature conditions (Chen *et al*., 2016). It has been also suggested that sublethal insecticide stress could select for higher temperature tolerance through their impact on the HSP pathways (Ge *et al*., 2013). Whether fungicides could act in a similar manner remains to be tested.

The variation in gene expression levels within each treatment was large in all the studied genes (Fig. 2) and hence we analyzed the overall treatment effect of the fungicide at the family level (Fig. 3). We found that the expression of all the investigated genes among the families show a more complex pattern. For example, whereas the overall expression of *Hsp70* showed no difference from the control samples at the higher concentration, there were differences among the families to different directions, which cancels out the overall effect. Similarly, even though the overall expression of *CYP9Z14v2* and *Ugt1* genes did not change under highest concentration and 24‐h exposure, there were some families which did respond to the fungicide. Similar variation in the expression levels of *Ugt* and 1 *CYP* gene was found among CPB adults under fungicide (chlorothalonil) treatment (Clements *et al*., 2018b), which highlights individual level variation in these detoxifying enzymes. Differences in the gene expression levels in different families under the same treatment group indicates that responses can vary within a beetle population and that there might be individual level genetic differences in responses to fungicides. It remains to be tested what are the underlying mechanisms for these differences.

## Conclusions

The field related concentrations of the fluazinam (0.12, 0.25 and 0.66 mg/L) did not have any significant negative effect on the behavior of a common nontarget pest species. Furthermore, there were no effects on larval survival even when individuals were exposed to fungicides for 3 d. However, we observed that this repeated exposure altered expression of the metabolic detoxification, insecticide resistance and stress tolerance genes. Since the expression levels differed among the families under different fungicide concentrations, this further suggests that there may be genetic differences in the tolerance to fungicides. Together, these results suggest that even though fluazinam only imposes minor effects in the short‐term, long‐term consequences should be also tested to fully understand how these widely used pesticides affect nontarget pest species and whether they will increase cross‐resistance with other pesticides.

## Disclosure

Authors declare they have no conflict of interest.
